# Idiopathic Premature Ventricular Contraction Catheter Ablation, Sedentary Population vs. Athlete’s Populations: Outcomes and Resumption of Sports Activity

**DOI:** 10.3390/jcm13071871

**Published:** 2024-03-24

**Authors:** Yari Valeri, Paolo Compagnucci, Giovanni Volpato, Lara Luciani, Eleonora Crepaldi, Francesco Maiorino, Quintino Parisi, Laura Cipolletta, Francesca Campanelli, Leonardo D’Angelo, Gemma Gaggiotti, Alessio Gasperetti, Andrea Giovagnoni, Antonio Curcio, Antonio Dello Russo, Michela Casella

**Affiliations:** 1 Cardiology and Arrhythmology Clinic, University Hospital “Ospedali Riuniti”, 60126 Ancona, Italy; paolo.compagnucci@ospedaliriuniti.marche.it (P.C.); giovanni.volpato@ospedaliriuniti.marche.it (G.V.); laraluciani95@gmail.com (L.L.); quintino.parisi@ospedaliriuniti.marche.it (Q.P.); laura.cipolletta@ospedaliriuniti.marche.it (L.C.); francesca.campanelli96@gmail.com (F.C.); leonardodangelo00@gmail.com (L.D.); gemmagag@alice.it (G.G.); antonio.dellorusso@gmail.com (A.D.R.); 2 Department of Biomedical Sciences and Public Health, Marche Polytechnic University, 60121 Ancona, Italy; 3 Allergology and Clinical Immunology, Reggio Emilia and Medena University, 41121 Modena, Italy; crepaldi.e98@gmail.com; 4 Military Hospital Center of Taranto, Cardiology Department, 74100 Taranto, Italy; f.p.maiorino@gmail.com; 5 Department of Cardiology, University Medical Center Utrecht, 3584 CX Utrecht, The Netherlands; alessio.gasperetti93@gmail.com; 6 Division of Medicine, Department of Cardiology, Johns Hopkins University, Baltimore, MD 21287, USA; 7 Department of Clinical, Special and Dental Sciences, Marche Polytechnic University, 60121 Ancona, Italy; andrea.giovagnoni@ospedaliriuniti.marche.it; 8 Division of Cardiology, Department of Medical and Surgical Sciences, Magna Graecia University, 88100 Catanzaro, Italy; antcurcio69@gmail.com

**Keywords:** premature ventricular contractions, right ventricular outflow tract, left ventricular outflow tract, catheter ablation, athletes, leisure-time athletes, agonist athletes, sports activity

## Abstract

There are no investigations about the outcomes of idiopathic PVC catheter ablation (CA) in athletes compared to the sedentary population. We conducted a prospective single-centre observational study. The primary and secondary procedural outcomes were the post-ablation reduction of premature ventricular contractions (PVCs) in an athletes vs. non-athletes group and in agonist vs. leisure-time athletes. The third was the evaluation of the resumption of physical activity and the improvement of symptoms in agonist and leisure-time athletes. From January 2020 to October 2022 we enrolled 79 patients with RVOT/LVOT/fascicular PVC presumed origin. The median percentage of decrease between the pre-procedure and post-procedure Holter monitoring in the non-athletes group was 96 (IQR 68–98) and 98 in the athletes group (IQR 92–99) (*p* = 0.08). Considering the athletes, the median percentage of decrease in the number of PVCs was 98 (IQR 93–99) and 98 (IQR 87–99), respectively, in leisure-time and agonistic athletes (*p* = 0.42). Sixteen (70%) leisure time and seventeen (90%) agonist athletes (*p* = 0.24) have resumed physical activity 3 months after PVC CA; among agonistic athletes, 59% have resumed competitive physical activity. Many leisure-time (88%) and agonist (70%) athletes experienced an improvement in symptoms after ablation. PVC CA was effective and safe in both groups, reducing symptoms and allowing a quick and safe return to sports activities in athletes.

## 1. Introduction

Premature ventricular contractions (PVCs) are commonly observed in healthy individuals undergoing 24-h ECG ambulatory monitoring, with a higher incidence in older age groups [1]. Studies comparing PVC prevalence in healthy athletes and sedentary individuals have shown no significant difference in the prevalence of ventricular arrhythmias between these populations [2]. Furthermore, the incidence of ventricular arrhythmias was found to be unrelated to the type, intensity, and duration of sports practice [2,3]. However, it is important to note that adolescents and young adults who participate in competitive sports have a higher risk of sudden cardiac death (SCD) compared to sedentary counterparts. Additionally, ventricular arrhythmias, including PVCs, can increase the risk of SCD during physical activity [4,5]. Therefore, European and American guidelines recommend specific evaluation with echocardiography, exercise testing, and 24-h ambulatory ECG monitoring, especially for athletes [6,7]. 

Cardiac Magnetic Resonance (CRM) with late gadolinium enhancement (LGE) is a second-line examination and the most accurate test to confirm or exclude the presence of structural heart disease (SHD) when there is a suspicion of SHD missed by echocardiography [8]. A workup using invasive electro-anatomical mapping can provide additional diagnostic information and improve the assessment of sports eligibility for athletes with ventricular arrhythmias [9,10,11].

Studies have shown that PVCs in athletes without apparent structural heart disease most commonly originate from the right or left ventricular outflow tract (RVOT/LVOT) or from the left fascicle [12,13,14]. RVOT/LVOT and fascicular PVCs are typically not associated with echocardiographic or CMR structural heart disease. During exercise testing, they tend to decrease or disappear at the peak of exercise and reappear during recovery [15]. Uncommon PVC morphologies are infrequent in athletes and are more commonly associated with LGE on CMR, which may be linked to underlying SHD [16].

Managing uncommon PVCs involves identifying potential underlying structural heart disease and implementing appropriate treatment. Both medical and ablative therapies can be used to address idiopathic PVCs [6]. 

Ablative approaches have demonstrated efficacy and safety, while medical therapy is generally poorly tolerated by young athletes. For individuals with symptomatic idiopathic PVCs and when the burden of high PVCs is associated with left ventricular dysfunction, an ablative approach is recommended [6]. 

This is especially important for those with infundibular and fascicular PVCs who may be restricted from participating in competitive sports. Additionally, ablative therapy, particularly for RVOT and fascicular PVCs, has been shown to be superior to medical therapy in several studies [4,6,17,18,19,20].

Catheter ablation (CA) of PVCs has demonstrated a high rate of procedural success both acutely and in the long-term, with a low overall complication rate. However, there is currently no research investigating the outcomes of PVC CA in athletes compared to sedentary individuals in patients without structural heart disease and with PVCs of benign morphology.

## 2. Materials and Methods

A prospective single-centre observational study was conducted to compare the efficacy and safety of radiofrequency CA of idiopathic benign morphology PVCs in athletes and sedentary individuals without SHD. The study also aimed to evaluate the return to physical activity after the ablative treatment in the athlete group.

The study adhered to institutional standards, national legal requirements, and the Helsinki Declaration for ethical standards. All patients provided written informed consent for the procedure.

### 2.1. Study Population

A total of 79 consecutive patients were enrolled between January 2020 and October 2022, all of whom had a class I or IIa indication for PVC CA according to current guidelines [6].

Medical examination, standard 12-lead ECG, 24-h ambulatory ECG monitoring, echocardiography, and maximal exercise testing were performed on all patients in accordance with European recommendations [6]. Additional instrumental evaluations were performed based on clinical judgement. In selected cases, CMR was also performed at the discretion of the physicians.

Inclusion criteria were:

- age >18 years,
- >2000/24 h PVCs at 24 h ambulatory ECG monitoring,
- PVC benign morphology (RVOT, LVOT, fascicular origin),
- preserved left ventricular ejection fraction (LVEF).

Exclusion criteria were:

- previous PVC CA,
- reduced LVEF (less than 50%),
- medical history of ischemic, hypertrophic, and right ventricular arrhythmogenic cardio-myopathy,
- family history of juvenile (<45 years) sudden death or hereditary cardiomyopathies,
- evidence of SHD at echocardiography or CMR,
- evidence of LGE in presumed area of origin of PVCs at CMR.

### 2.2. Study Protocol (Design and Setting)

Enrolment was limited to patients who met the inclusion criteria. Two expert cardiologists evaluated the presumed origin of ventricular extrasystole in relation to the 12-lead ECG characteristics. In case of disagreement, a third cardiologist was consulted. Comprehensive physical activity and medical histories were obtained.

The patients were classified into two groups: group 1 consisted of athletes, defined as individuals who engage in regular exercise training and participate in official sports competitions, regardless of age or amateur/professional status; and group 2 consisted of non-athletes. The group of athlete patients was subsequently divided into competitive and leisure-time athletes. Competitive athletes are highly trained with a greater emphasis on performance and winning, typically at the high school, college, and master club level, and usually exercise for more than six hours per week. Leisure-time athletes engage in sports for pleasure and leisure-time activity, and usually exercise for more than four hours per week [18].

In addition, the study evaluated the physical activity level of the patients prior to ablation. This included assessing the intensity of their exercise, the type of sport they participated in, the number of weekly training sessions, the duration of weekly training hours, and any limitations in physical activity due to PVCs.

All patients underwent a medical examination, standard 12-lead ECG, 24-h ambulatory ECG monitoring, echocardiography, and maximal exercise testing. After obtaining written informed consent, all patients underwent radiofrequency CA.

The patients’ PVCs were determined to not have an ischemic origin based on their morphology (RVOT, LVOT, fascicular), absence of structural heart disease on both the 12-lead ECG and echocardiogram, lack of typical ischemic symptoms reported in the patients’ histories, benign behaviour during the exercise test, and absence of signs indicating inducible myocardial ischemia during the same test.

### 2.3. Radiofrequency Catheter Ablation

The procedures for both athletes and non-athletes were carried out by an experienced electrophysiologist with at least 10 years of experience in PVC CA.

Sedation or general anaesthesia was not administered at the start of the procedure to avoid compromising the presence of PVCs. Operators used conscious sedation with i.v. dexmedetomidine and fentanyl or simple analgesia with fentanyl at their discretion after mapping.

Following ultrasound-guided femoral vein catheterization using the Seldinger technique, an ablation catheter and decapolar catheter were introduced for coronary sinus mapping.

The use of intra-cardiac echocardiography was at the physician’s discretion. For left-sided procedures, the retro-aortic approach was preferred. If necessary, a transseptal puncture was performed, followed by replacement of the SL0 introducer with the AgilisTM NxT steerable introducer. When accessing the left ventricle prior to transseptal puncture or retro-aortic access, a bolus of unfractionated heparin (UFH) at a dose of 100 IU/kg was administered. The UFH infusion was then continued until the conclusion of the procedure, maintaining an activated clotting time of over 300 s.

If PVCs were absent or infrequent at the time of CA, various methods were used to induce them. Initially, intravenous infusion of isoprenaline was administered. If this approach was ineffective, a programmed atrial/ventricular pacing study was conducted using up to three extrastimuli and burst pacing. As a last resort, i.v. caffeine infusion was used as an alternative method. Pacemapping was performed to assess whether the QRS morphology at the earliest site of activation matched with the morphology of spontaneous PVCs. CA was performed at the location showing the best pace-match, typically over 95%. Furthermore, as PVCs causes a position shift in 3D mapping systems due to motion of the heart within the 3D coordinate system, we used the LAT-hybrid module. The LAT-hybrid module allows correcting for the three-dimensional positional shift caused by extrasystolic contraction compared to the map performed in sinus rhythm.

**Figure 1:**
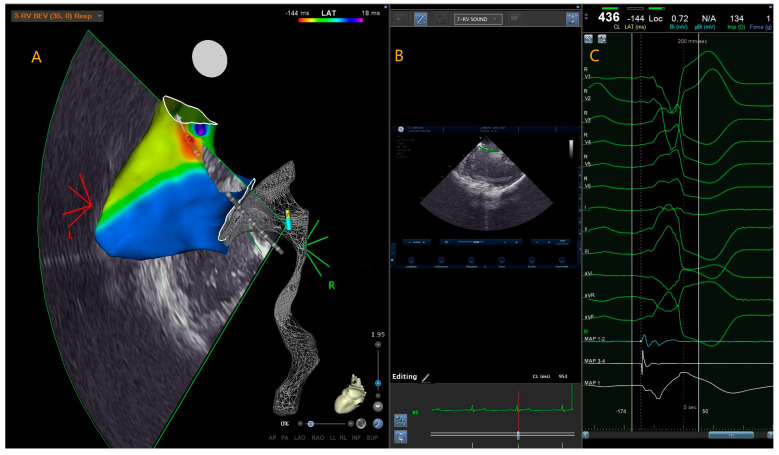
This is a local activation time map of a 36-year-old leisure-time athlete with idiopathic premature ventricular contractions originating from the right ventricular outflow tract. The colorimetric map indicates that the primary site of onset of premature ventricular contractions is the posterior right ventricular outflow tract. In this region, the ablator catheter is visible in transparency, with the tip at the level of the region of interest (**A**). Intracardiac echocardiography and the CARTOSOUND^®^ Module with SOUND-STAR^®^ can be used to integrate the electro-anatomical map with the intracardiac echocardiographic map. This allows for visualisation of the catheter and its tip position using ICE (**B**). The bipolar ablator catheter signal is more advanced than the surface QRS signal, and the unipolar signal is represented by a QS signal (**C**). PVC (premature ventricular complex); RVOT (right ventricular outflow tract); ICE (intra cardiac echocardiography).

Anatomical mapping data were collected using a 3D mapping system (CARTO 3, Bio-sense Webster) and a contact force mapping-ablation catheter (ThermoCool SmartTouch, Biosense Webster Inc., Minneapolis, MA, USA). The activation mapping was performed in each case: the PVC origin site was defined as the earliest site of local ventricular activation preceding the onset of the QRS wave by at least 30 ms on the surface ECG and with a QS signal in unipolar mapping (Figure 1).

Radiofrequency energy pulses were delivered using a 3.5 mm tip irrigated ablation catheter (ThermoCool SmartTouch™, Biosense Webster Inc., Minneapolis, MA, USA) at a power of 35 to 50 W with a target Ablation Index of 550 to 630. If the PVCs were eliminated, an additional pulse of radiofrequency was delivered at the same site. If PVCs persisted, mapping was continued to locate an optimal target site [21,22].

### 2.4. Follow-Up

Amiodarone and flecainide were discontinued in all patients in whom ablation was acutely effective. Beta-blockers were continued or suspended based on the doctor’s clinical decision.

Both athlete and non-athlete patients were prohibited from practicing sports for at least two weeks after the procedure. For the first month, it is recommended to gradually restore sporting activity, followed by resuming full physical activity approximately 45 days after the procedure.

All patients underwent a 24-h Holter ECG monitoring 3 to 6 months after the index procedure. Patients were evaluated with an in-office visit after 9 to 15 months, including physical examination and a 12-lead ECG. Patients who could not schedule an in-office check-up were examined through telephonic monitoring.

Patients who reported symptoms such as palpitations, dizziness, or syncope, or whose 12-lead ECG showed the presence of PVCs during follow-up, were advised to contact their referring physician for an evaluation with a 12-lead 24-h Holter monitor.

### 2.5. Study Endpoint

The study’s primary procedural outcome was the post-ablation reduction of PVCs in both athletes and non-athletes groups. This was evaluated using 12-lead 24-h ECG Holter monitoring, performed 3–6 months after ablation.

The secondary procedural outcome was the post-ablation reduction of PVCs in both competitive and leisure-time athletes.

The evaluation of the resumption of physical activity and the subjective improvement of symptoms in both competitive and leisure-time athletes was the third procedural outcome.

### 2.6. Statistical Analysis

Continuous variables were checked for normality with the Shapiro-Wilk test and presented as mean and standard deviation (SD) if normally distributed, or as median and interquartile range (IQR: 25th percentile, 75th percentile) if non-normally distributed. Categorical variables are given as count and percentage (%). Comparisons between groups were made with chi squared test, Student’s t test, and Mann-Whitney U test. *p* < 0.05 was considered statistically significant. The software Statistical Package for Social Science v25 (SPSS^®^ Inc., IBM^®^, NY, USA) was used for statistical analyses.

## 3. Results

### 3.1. Patient Population

The baseline characteristics of all patients have been summarized in Table 1.

The study enrolled 84 patients with presumed RVOT/LVOT/fascicular origin of PVCs based on analysis of the 12-lead electrocardiogram. Five patients were later excluded as electroanatomic mapping did not confirm PVC origin (4 LV summit and 1 mitro-aortic continuity locations). The electrocardiographic criteria used to determine PVC origin are summarized in the Supplementary Materials.

Out of the 79 patients who were enrolled, the majority had no cardiovascular risk factors and the dimensions and functions of both the right and left ventricles were within normal limits. More than half of the enrolled patients were already on beta-blocker therapy, while a smaller proportion were receiving treatment with flecainide or amiodarone. The median number of PVCs in the enrolled patients was very high (median 19,600, IQR 11,100–30,600). All 79 presumed origins from the 12-lead ECG were confirmed using electro-anatomical mapping. Of these, 57 (72%) were from the RVOT, 19 (24%) were from LVOT, and 3 (4%) were fascicular.

Out of the 79 patients enrolled, 23 (30%) did not undergo MRI, while 56 (70%) underwent the examination to exclude potential structural heart conditions more precisely. Among the 56 MRIs conducted, 37 yielded negative results for both structural heart issues (including wall thickness, ventricular chamber volumes, and wall motion abnormalities) and the detection of LGE areas. However, the remaining 19 MRI scans revealed the presence of areas with LGE but consistently showed no signs of structural heart problems. In all cases, MRIs demonstrated limited areas of LGE (<20%) in the left ventricle, in regions unrelated to the presumed PVC origin. In this study, it was found that LGE areas were located exclusively in the pericardium in five patients, indicating previous pericarditis. In three patients, the areas were found in the anterior/junctional septal area, while in eight and three patients, the areas were located within the intramyocardial region along the basal posterolateral wall and within the anterolateral wall, respectively, indicating myocardial inflammation.

### 3.2. Comparison of Athlete and Non-Athlete Groups

The study involved 79 patients, divided into two groups: non-athletes (n = 37; mean age 53.2 ± 11.2 years; male 24, 64%) and athletes (n = 42; mean age 39 ± 12.8 years; male 27, 64%).

The non-athlete group had a statistically higher mean age than the athlete group. Additionally, the incidence of cardiovascular risk factors was slightly higher in the non-athlete group. There were no significant differences observed in the use of antiarrhythmic medications between the two groups, except for beta-blockers, which were less commonly used in athletes (*p* < 0.001). Additionally, there were no statistically significant differences in echocardiographic characteristics or PVC burden at baseline 24-h ambulatory ECG monitoring between the two groups.

The leisure-time subgroup consisted of 23 patients (42 ± 12.5 years; 13 male, 57%), while the competitive subgroup included 19 patients (32.9 ± 12.9 years; 14 male, 74%). In the athletes group, there were two subgroups: leisure-time and competitive. Competitive athletes were significantly younger than leisure-time athletes. There was no statistically significant difference between the two subgroups in terms of echocardiographic features and antiarrhythmic drug therapy.

Concerning the types of sports practiced by leisure-time athletes: 43% engaged in endurance sports, 40% in mixed sports, and 7% in power sports; concerning intensity, 48% participated in low-intensity sports, and 52% in moderate-intensity sports.

Regarding competitive athletes, the most frequently practiced sports were endurance sports (n = 9, 47%) and mixed sports (n = 9, 47%), followed by power sports (n = 1, 5%); regarding intensity, sports were practiced at high intensity in 79% of cases and at moderate intensity in 21% of cases [18].

As expected, competitive athletes had a statistically significant greater number of weekly workouts and weekly training hours compared to the leisure-time athletes (three [IQR 2–3] vs. four [IQR 3–5] workouts per week and five [IQR 3–6] vs. nine [IQR 7–12] hours per week). However, no significant differences in PVC-induced symptoms were observed between the two subgroups.

The baseline characteristics of athlete subgroups have been summarized in Table 2.

### 3.3. Procedural Data

The table presents all procedural data, with few exceptions showing no statistically significant differences between the groups.

Activation mapping was conducted on all patients, including both athletes and non-athletes. In some cases, drug infusions such as caffeine and isoproterenol (20–30% of cases) and stimulation protocols (approximately 10% of cases) were used to facilitate PVC induction. In most cases, pace-mapping manoeuvres were used, resulting in excellent matching values (>95%). Mapping procedures were consistently performed using the ablator catheter, except in rare cases where multi-polar catheters were necessary. The median maximum advancement of the ablator catheter signal on the surface QRS was 30 ms.

After precisely identifying the region of interest, a limited number of deliveries and a constrained total delivery time were required. The median number of VisiTags per procedure was six, with an ablated area of 2.2 cm^2^ and a total delivery duration of approximately 175 ms. The maximum achieved AI was consistently greater than 600. Fluoroscopy times were notably shorter in athlete patients compared to non-athletes, particularly in competitive patients (refer to Table 3).

In non-athlete patients, the median number of 24-h PVCs was 17,000 (IQR 10,000–30,000), whereas in athletes, it was 18,750 (IQR 10,000–30,700). The most frequent origin site for PVCs was the RVOT, which was observed in 59 out of 79 patients (72%), with 80% in athletes and 62% in non-athletes. In both groups, the anterior region of the RVOT was the most affected (40% in group 1 and 38% in group 2), followed by the septal and posterior regions (26% vs. 32% and 34% vs. 30%, respectively).

**Figure 2:**
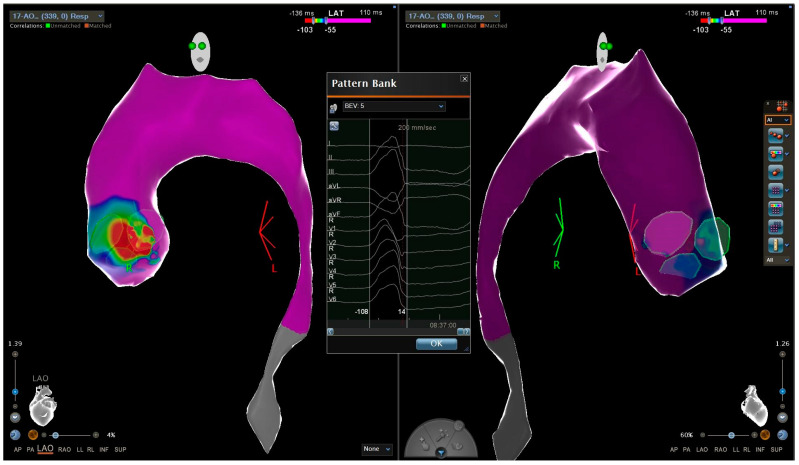
This is a local activation time map of a 24-year-old sedentary man with idiopathic premature ventricular contractions originating from the left ventricular outflow tract. The colorimetric map indicates that the primary site of onset of premature ventricular contractions is the commissure region between the left and non-coronary aortic cusp. PVC (premature ventricular complex); LVOT (left ventricular outflow tract).

Conversely, ablation of LVOT PVCs was more frequently performed in non-athlete patients compared to athletes (32% vs 17%) (see Figure 2).

Acute efficacy was achieved in 86% of non-athletes and 90% of athletes, defined as the absence of PVCs during the first 30 min of ECG monitoring after CA and/or at ECG telemetry monitoring during the first 24 h after CA.

Only four minor vascular complications occurred, all of which were pseudo-aneurysms, and none required surgery (refer to Table 4 and Table 5).

Amiodarone was discontinued in all patients. Flecainide was discontinued in all patients in whom ablation was acutely effective. In patients in whom ablation was not acutely effective, the decision to continue or discontinue flecainide was made by the doctor. Flecainide was continued in three non-athlete patients and two athlete patients. The decision to continue or suspend beta-blockers was also made by the doctor based on clinical judgement. The study was conducted on fourteen patients, ten of whom were non-athletes and four of whom were athletes (three leisure-time and one competitive).

Follow-up was conducted at 9–15 months, with 77% of patients visiting in person and the remaining 23% contacted by phone. Table 6 summarizes the specifics of the follow-up visit for patients in whom the ablation was acutely effective, while Table 7 summarizes the same for patients in whom the ablation was not acutely effective.

Prior to cardiac ablation, the median number of PVCs in patients who engaged in leisure-time physical activity was 26,500 (IQR 11,000–26,700), while in competitive athletes it was 12,000 (IQR 10,000–27,000). The most common site of origin for PVCs was the RVOT, followed by the LVOT and fascicular origin. Statistical analysis did not reveal any significant differences in the site of origin between the leisure-time and competitive groups.

### 3.4. Outcomes

All patients underwent 12-lead Holter ECG monitoring 3–6 months after the procedure. The mean follow-up time until 24-h Holter ECG monitoring was 4.9 (±0.8) months. The median number of PVCs over 24 h was 1000 (IQR 341–4916) in non-athlete patients and 300 (IQR 167–851) in athletes. The study evaluated the percentage decrease in PVC number between pre-procedure and post-procedure 12-lead Holter monitoring for each patient. The median percentage of decrease in the non-athlete group was 96% (IQR 68–98), and in the athlete group, it was 98% (IQR 92–99). There was no statistically significant difference in the reduction of PVC percentage after the ablation procedure between the non-athlete and athlete groups (*p* = 0.08). The median burden of PVCs during 12-lead Holter ECG monitoring was 300 (IQR 230–800) and 260 (IQR 160–1000) for leisure-time and agonistic patients, respectively. The median percentage decrease in PVCs between pre-procedure and post-procedure 12-lead Holter monitoring was 98% (IQR 93–99) and 98% (IQR 87–99) for leisure-time and agonistic patients, respectively. There was no statistically significant difference in the percentage reduction of PVCs after the ablation procedure between the leisure-time and agonistic groups (*p* = 0.42) (refer to Table 8).

### 3.5. Post-Ablation Sport Activity

The study compared pre-ablation values with corresponding post-ablation values to evaluate the resumption of physical activity after the procedure.

At 3 months after PVC CA, 70% (n = 16) of leisure-time athletes and 90% (n = 17) of competitive athletes had resumed physical activity (*p* = 0.24). Among agonistic athletes, 59% (n = 10) had resumed competitive physical activity. Out of the nine patients (41%) who had not resumed competitive sports activities at 3 months, seven (77%) reported resuming competitive activities after approximately a year during the follow-up. However, two patients (23%), one of whom did not have successful ablation, had not resumed competitive sports activities and instead continued with non-competitive sports activities.

There were no statistically significant differences in the number of pre- and post-procedural training sessions or weekly training hours among athletes who resumed physical activity, regardless of whether they were competitive or leisure-time athletes.

Similarly, no statistically significant differences were observed between competitive and leisure-time athletes in terms of the number of workouts (*p* = 0.97 and *p* = 0.15) and weekly training hours (*p* = 0.92 and *p* = 0.10) before and after the procedure.

## 4. Discussion

This study evaluates the efficacy of CA for idiopathic PVCs in both non-athletes and athletes, including competitive and leisure-time athletes. Additionally, the study examines the resumption of physical activity and subjective improvement of symptoms post-ablation in competitive and leisure-time athletes.

Our findings confirm a high efficacy rate of CA in treating idiopathic PVCs, not only among non-athletes but also in athletes. Athletes who undergo CA exhibit a notable rate of resumption of physical activity. After ablation, athletes experience a significant improvement in exercise tolerance, reflecting the positive impact of this effective procedure on their overall fitness level.

According to the guidelines of the European Society of Cardiology (ESC), CA is a recommended therapeutic strategy for treating symptomatic patients with presumed idiopathic premature ventricular contractions (PVCs), particularly those originating from the RVOT and left fascicles. The literature reports a high success rate of CA for idiopathic PVCs, with rare complications, particularly for those originating from the RVOT and the fascicles [6]. In this series, we fully analysed patients who underwent CA for apparently idiopathic PVCs. We then categorised the patients into two groups: non-athletes and athletes. Within the athletes group, we further classified them into competitive and leisure-time for further investigation.

As expected, non-athletes had a significantly higher median age than athletes. Similarly, the median age of leisure-time athletes was greater than that of competitive athletes. However, there were no other noticeable differences in terms of clinical characteristics and echocardiographic features between the two groups.

The use of antiarrhythmic drugs before CA was less common among athletes than non-athletes. It is worth noting that beta-blocker therapy, which is the most frequently prescribed medication in the non-athlete population, was almost always avoided in the athlete group. There are several reasons for this discrepancy. Firstly, introducing beta-blocker drugs into athlete therapy is more challenging due to their typically higher vagotonic tone and lower resting heart rate. Secondly, such therapy can potentially impact their physical sports performance, which is less desirable for athletes. Furthermore, athletes often opt for ablation at an earlier stage compared to non-athletes, and sometimes they do so without testing any medical therapy in order to obtain consent for resuming sports, which is a priority for them.

Although there was no statistically significant difference, PVCs from LVOT tended to occur more frequently in non-athletes. It is interesting to note that the non-athlete patients tended to be older and have a higher prevalence of cardiovascular risk factors. As Kurshunov et al. [23] suggested, these factors are associated with a higher occurrence of LVOT PVCs.

The number of PVCs detected during the 24-h pre-procedural Holter ECG monitoring was found to be remarkably high in both the athletes and non-athletes groups, with a median count of 17,000 and 18,750, respectively. However, when comparing athletes, the competitive athletes showed a lower number of PVCs compared to leisure-time athletes, although this difference was not statistically significant. This finding can potentially be explained by the fact that competitive athletes undergo regular sports check-ups, enabling the early detection of PVCs and prompt consideration of ablative options. Furthermore, athletes requiring agonistic fitness certification undergo CA in earlier stages.

The results of CA for PVCs were consistently excellent in all patient groups, as evidenced by the acute efficacy of CA and the number of PVCs recorded in the 12-lead post-procedure Holter ECG. These data confirm that CA is an effective therapeutic option for treating idiopathic PVCs, even among athletes and individuals engaged in sports.

Many athletes affected by PVCs experience symptoms at rest and/or during physical activity, which often leads to suspension or significant decrease in their participation in physical activities, as they are unable to maintain their competitive fitness. This study found that around 80% of leisure-time athletes and 85% of competitive athletes experienced a reduction in physical activity either at the time of diagnosis or shortly before it.

Regarding the resumption of physical activity after ablation, a significant percentage of leisure-time athletes (70%) and competitive athletes (90%) were able to resume physical activities. This suggests that the interventional ablative option does not discourage patients from engaging in physical activity, but rather encourages their recovery. However, around 40% of patients who previously participated in competitive sports and resumed physical activity after CA did not return to the same level of intensity.

Regarding the frequency and duration of weekly workouts, there were no statistically significant differences, but both the number of workouts and training hours showed a numerical decrease after the ablative procedure. It is important to note that these findings may have been influenced by the restrictions imposed by the SARS-CoV-2 pandemic and the gradual resumption of physical exercise, which eventually reached a plateau after a longer latency time.

Approximately 33% (n = 14) of athletes with a diagnosed high burden of PVCs reported symptoms during physical exertion before CA. The group consisted of nine leisure-time athletes and five competitive patients. These symptoms included easy fatigue, dyspnea, and palpitations, which affected their physical activity.

Among the patients who resumed physical activity (n = 33), 88% (n = 14) of leisure-time athletes and 70% (n = 12) of competitive athletes reported a significant improvement in functional capacity and post-ablation performance.

These data are unexpected: there are more individuals who experienced an improvement in physical performance during exercise compared to athletes who reported symptoms before CA.

This seemingly paradoxical result can be explained in two main ways:

- Patients with a high burden of PVCs who claim to be asymptomatic may have developed compensatory mechanisms to cope with their symptoms. They may have engaged in physical activity below their maximum potential, adapting to their chronic symptoms and thus delivering suboptimal performances.
- The interventional ablative procedure and 24-h post-procedure ECG Holter monitoring, which documented a decrease in PVCs, may have had a placebo effect on patients, leading to their perception of improved symptoms and performance.

### Limitations

The study has several limitations.

Firstly, due to the small sample size, type II error cannot be excluded.

Secondly, the patients’ symptoms and improvement in physical activity were collected subjectively without the use of any questionnaire or objective methods. The use of cardiopulmonary exercise testing could have provided more accurate data for exercise capacity.

Not all patients underwent cardiac MRI to accurately exclude structural heart disease.

Lastly, the study focused on relatively older athletes, predominantly falling into the masters category, regarding mean age data. It would be interesting to examine the results of PVCs ablation among younger athletes and age-matched non-athletes as well.

## 5. Conclusions

To our knowledge, this is the first study to compare the efficacy and safety of CA of benign PVCs in athletes and non-athletes. Our study demonstrated that CA was effective and safe in both groups, reducing symptoms and enabling a prompt and safe return to sports activities for athletes.

## Figures and Tables

**Table 1:** Baseline non-athletes’ and athletes’ characteristics. Values are counts, mean (SD), or median (first quartile, third quartile). SD (standard deviation); N (number); LVEF (left ventricular ejection fraction); IQR (interquartile range); TAPSE (tricuspid annular plane systolic excursion); RVD1 (right ventricle diameter); ILVEVD (index left ventricle end diastolic volume); EGFR (estimated glomerular filtration rate).

	GENERAL POPULATION (N = 79)	NON-ATHLETES (N = 37)	ATHLETES (N = 42)	*p* VALUE
AGE (YEARS)—MEAN (SD)	46 (14.5)	53.2 (11.2)	39 (12.8)	*p* < 0.0001
MALE SEX—N (%)	51 (65)	24 (64)	27 (64)	*p =* 0.9
LVEF %—MEDIAN [IQR]	56.5 (53—61)	56 (50–60.5)	55 (54–60)	*p =* 0.37
TAPSE (MM)—MEAN (SD)	24.3 (3.1)	24.2 (2.5)	24.8 (3.6)	*p =* 0.27
RDV1 (MM)—MEAN (SD)	36.2 (4.8)	36 (5.3)	36.7 (4.4)	*p =* 0.2
ILVEDV (ML/M2)—MEAN (SD)	63.3 (13.8)	63.5 (15.3)	63.2 (11. 5)	*p =* 0.46
HYPERTENSION—N (%)	12 (15)	8 (21)	4 (9)	*p =* 0.23
DYSLIPIDEMIA—N (%)	18 (23)	14 (37)	4 (9)	*p =* 0.006
DIABETES MELLITUS—N (%)	2 (3)	2 (5)	0 (0)	*p =* 0.9
EGFR < 45 ML/MIN/M2—N (%)	1 (1)	1 (2)	0 (0)	*p =* 0.9
PRE-ABLATION AAD THERAPY—N (%)	42 (53)	28 (76)	14 (33)	*p* < 0.001
-BETA-BLOCKER—N (%)	31 (39)	23 (62)	8 (19)	*p* < 0.001
-FLECAINIDE—N (%)	12 (15)	7 (19)	5 (12)	*p* = 0.58
-AMIODARONE—N (%)	6 (7)	5 (13)	1 (2)	*p* = 0.15
N° WORKOUTS PER WEEK—MEDIAN [IQR]			3 (2.75–4)	
WORKOUTS HOURS PER WEEK—MEDIAN [IQR]			6 (4.75–9)	
SYMPTOMS DURING ACTIVITY—N (%)			14 (33)	

**Table 2:** Baseline leisure-time and agonist athletes characteristics. Values are counts, mean (SD), or median (first quartile, third quartile). SD (standard deviation); N (number); LVEF (left ventricular ejection fraction); TAPSE (tricuspid annular plane systolic excursion); RVD1 (right ventricle diameter); ILVEVD (index left ventricle end diastolic volume); EGFR (estimated glomerular filtration rate).

	LEISURE-TIME (N = 23)	COMPETITIVE (N = 19)	*p* VALUE
AGE (YEARS)—MEAN (SD)	44 (12.5)	32.9 (12.9)	*p* = 0.004
MALE SEX—N (%)	13 (57%)	14 (74%)	*p* = 0.4
LVEF %—MEAN (SD)	56.7 (6.5)	56.6 (5)	*p =* 0.46
TAPSE (MM)—MEAN (SD)	25.2 (4.2)	24.2 (2.7)	*p =* 0.19
RDV1 (MM)—MEAN (SD)	36.4 (5)	37.1 (3.3)	*p =* 0.5
ILVEDV (ML/M2)—MEAN (SD)	61.9 (12.8)	64.8 (9.3)	*p =* 0.21
HYPERTENSION—N (%)	3 (13)	1 (5)	*p =* 0.74
DYSLIPIDEMIA—N (%)	2 (9)	2 (11)	*p =* 0.74
DIABETES MELLITUS—N (%)	0	0	
EGFR < 45 ML/MIN/M2—N (%)	0	0	
PRE-ABLATION AAD THERAPY– N (%) -BETA-BLOCKER—N (%) -FLECAINIDE—N (%) -AMIODARONE—N (%)	7 (30) 4 (17) 2 (9) 1 (4)	7 (36) 4 (21) 3 (16) 0	*p =* 0.66 *p =* 0.92 *p =* 0.81 *p =* 0.9
N° WORKOUTS PER WEEK—MEDIAN [IQR]	3 (2–3)	4 (3–5)	*p =* 0.003
WORKOUTS HOURS PER WEEK—MEDIAN [IQR]	5 (3–6)	9 (7–12)	*p*< 0.001
SYMPTOMS DURING ACTIVITY—N (%)	9 (39)	5 (26)	*p =* 0.58
DECREASE OR INTERRUPTION OF PHYSICAL ACTIVITY—N (%)	18 (80%)	16 (85%)	*p =* 0.92

**Table 3:** Procedural data. Values are counts, mean (SD), or median (first quartile, third quartile). N (number); IQR (interquartile range); RF (radiofrequency); AI (ablation index); SD (standard deviation); SVP (programmed ventricular stimulation).

	NON-ATHLETES (N = 37)	ATHLETES (N = 42)	*p* VALUE	LEISURE-TIME (N = 23)	COMPETITIVE (N = 19)	*p* VALUE
ACTIVATION MAP—N (%)	37 (100)	42 (100)	*p* ns	23 (100)	19 (100)	*p* ns
PATTERN MATCHING—N (%)	34 (91)	38 (90)	*p* = 0.82	20 (86)	18 (94)	*p =* 0.74
PASO VALUE—MEDIAN [IQR]	97 (97–98)	97 (95–98)	*p* = 0.23	97 (95–97)	97 (96–98)	*p =* 0.65
MAXIMUM SIGNAL ADVANCE (MSEC)—MEDIAN [IQR]	30 (29–35)	30 (28–38)	*p* = 0.85	30 (29–37)	30 (28–38)	*p =* 0.88
STIMULATION PROTOCOL—N (%)	4 (10)	3 (7)	*p* = 0.67	1 (5)	2 (10)	*p =* 0.86
CAFFEINE/ISOPROTENEROL—N (%)	8 (21)	11 (26)	*p* = 0.83	4 (17)	7 (36)	*p =* 0.28
MULTIPOLAR CATHETER—N (%) ABLATOR CATHETER—N (%)	4 (10) 37 (100)	3 (7) 42 (100)	*p* = 0.67 *p* ns	1 (5) 23 (100)	2 (10) 19 (100)	*p =* 0.86 *p* ns
RF TIME (SEC)—MEDIAN [IQR]	175 (143–240)	173 (146–246)	*p =* 0.59	195 (155–310)	167 (145–230)	*p =* 0.25
VISITAG—N (%)	6 (5.5–7.5)	6 (5–9)	*p* = 0.48	6 (5–9)	5 (5–6)	*p =* 0.09
MAX AI—MEAN (SD) MEAN AI—MEAN (SD)	621 (21) 534 (57)	610 (24) 546 (41)	*p* = 0.16 *p =* 0.23	610 (18) 560 (32)	605 (30) 535 (42)	*p =* 0.25 *p =* 0.02
ABLATION AREA (CM2)—MEDIAN [IQR]	2.15 (1.6–3)	2.2 (1.8–2.8)	*p* =0.77	2.1 (1.8–2.8)	2.25 (1.7–2.8)	*p =* 0.7
PROCEDURE DURATION (MIN)—MEDIAN [IQR]	85 (75–135)	85 (75–140)	*p* = 0.66	90 (76–125)	75 (67–147)	*p =* 0.24
FLUOROSCOPY TIME (MIN)—MEDIAN [IQR]	12.5 (8–22)	8.2 (5–10.5)	*p =* 0.02	8.5 (6–14)	6 (4–8)	*p =* 0.02
SVP—N (%)	25 (67)	29 (69)	*p =* 0.9	14 (60)	15 (79)	*p =* 0.35

**Table 4:** Procedural data information. Values are counts, mean (SD), or median (first quartile, third quartile). RVOT (right ventricular outflow tract); N (number); LVOT (left ventricular outflow tract); PVC (premature ventricular complex); IQR (interquartile range).

	NON-ATHLETES (N = 37)	ATHLETES (N = 42)	*p* VALUE
SITE OF ORIGIN			
- RVOT– N (%)	23 (62)	34 (80)	*p =* 0.10
*_SEPTAL RVOT*—N (%)	14 (60)	23 (67)	*p =* 0.8
_FREE WALL RVOT—N (%)	9 (40)	11 (32)	*p =* 0.8
- LVOT– N (%)	12 (32)	7 (17)	*p =* 0.16
- FASCICULAR—N (%)	2 (5)	1 (3)	*p =* 0.91
PRE-PROCEDURE N° PVC—MEDIAN [IQR]	17,000	18,750	*p =* 0.12
	(10,000–30,000)	(10,000–30,700)	
ACUTE EFFECTIVENESS—N (%)	32 (86)	38 (90)	*p =* 0.83
MAJOR COMPLICATIONS—N (%)	0 (0)	0 (0)	
MINOR COMPLICATIONS—N (%)	2 (5)	2 (4)	*p =* 0.7
POST-PROCEDURE N° PVC—MEDIAN [IQR]	1000	300	*p =* 0.09
	(341-4916)	(167–851)	

**Table 5:** Procedural data information. Values are counts, mean (SD), or median (first quartile, third quartile). RVOT (right ventricular outflow tract); N (number); LVOT (left ventricular outflow tract); PVC (premature ventricular complex); IQR (interquartile range).

	LEISURE-TIME (N = 23)	COMPETITIVE (N = 19)	*p* VALUE
SITE OF ORIGIN - RVOT– N (%) *_SEPTAL RVOT*—N (%) _FREE WALL RVOT—N (%) - LVOT– N (%) - FASCICULAR– N (%)	18 (78) 12 (67) 6 (33) 4 (17) 1 (4)	16 (84) 11 (68) 5 (32) 3 (16) 0	*p =* 0.92 *p =* 0.81 *p =* 0.81 *p =* 0.78 *p =* 0.9
N° PRE-PROCEDURE PVC—MEDIAN [IQR]	26,500 (11,000–26,700)	12,000 (10,000–27,000)	*p =* 0.06
ACUTE EFFECTIVNESS—N (%)	20 (87)	18 (95)	*p =* 0.74
MINOR COMPLICATIONS—N (%)	1 (4)	1 (5)	*p =* 0.55
N° POST-PROCEDURE PVC—MEDIAN [IQR]	300 (230–800)	260 (160–1000)	*p =* 0.29

**Table 6:** Follow-up for patients in whom the ablation was acutely effective. Values are counts, mean (SD), or median (first quartile, third quartile). N (number); PVC (premature ventricular complex); ECG (electrocardiogram); IQR (interquartile range).

	NON-ATHLETES (N = 32)	ATHLETES (N = 38)	*P* VALUE	LEISURE-TIME (N = 20)	COMPETITIVE (N = 18)	*p* VALUE
ON SITE FOLLOW-UP—N (%)	24 (75)	30 (79)	*p* = 0.88	14 (70)	16 (89)	*p =* 0.30
CONTACT BY PHONE—N (%)	8 (25)	8 (21)	*p* = 0.91	6 (30)	2 (11)	*p =* 0.30
NO SYMPTOMS—N (%)	32 (100)	31 (100)	*p* ns	20 (100)	18 (100)	*p* ns
PRESENCE OF PVCS ON FOLLOW-UP ECG—N (%)	2 (6)	2 (5)	*p* = 0.73	1 (5)	1 (5)	*p =* 0.51
24-H-HOLTER ECG—N (%)	15 (46)	24 (63)	*p* = 0.26	11 (55)	13 (72)	*p =* 0.44
PVC NUMBER ON HOLTER—MEDIAN [IQR]	600 (300–600)	475 (230–500)	*p* = 0.29	500 (230–500)	450 (275–450)	*p* = 0.81

**Table 7:** Follow-up for patients in whom the ablation was not acutely effective. Values are counts, mean (SD), or median (first quartile, third quartile). N (number); PVC (premature ventricular complex); ECG (electrocardiogram); IQR (interquartile range).

	NON-ATHLETES (N = 32)	ATHLETES (N = 38)	LEISURE-TIME (N = 20)	COMPETITIVE (N = 18)
ON SITE FOLLOW-UP—N (%)	3 (60)	2 (50)	1 (33)	1 (100)
CONTACT BY PHONE—N (%)	2 (40)	2 (50)	2 (67)	0 (0)
NO SYMPTOMS—N (%)	3 (60)	3 (75)	2 (67)	1 (100)
PRESENCE OF PVCS ON FOLLOW-UP ECG—N (%)	3 (60)	1 (25)	1 (33)	0 (0)
24-H-HOLTER ECG—N (%)	4 (80)	3 (75)	2 (67)	1 (100)
PVC NUMBER ON HOLTER	6000, 26,000, 29,000, 34,000	304,200, 14,800	4200, 14,800	30

**Table 8:** Post-ablation sport activity. Values are counts, mean (SD), or median (first quartile, third quartile). N (number); IQR (interquartile range).

	LEISURE-TIME (N = 23)	COMPETITIVE (N = 19)	*p* VALUE
RESUMPTION OF PHYSICAL ACTIVITY—N (%)	16 (70)	17 (90)	*p* = 0.24
RESUMPTION OF COMPETITIVE PHYSICAL ACTIVITY—N (%)		10 (59)	
WORKOUTS PER WEEK—N (%)	2.5 (2–4)	3 (2–4)	*p =* 0.3
WORKOUT HOURS PER WEEK—MEDIAN [IQR]	4 (3.25–6)	7 (6–8.5)	*p =* 0.007
IMPROVEMENT OF SYMPTOMS DURING SPORT ACTIVITY— N (%)	14 (88%)	12 (70%)	*p =* 0.44

## Data Availability

The data presented in this study are available on request from the corresponding author due to privacy and legal reasons.

## Supplementary Materials

The following supporting information can be downloaded at: https://www.mdpi.com/article/10.3390/jcm13071871/s1. See Supplementary Materials [24,25,26].
