# Supplementary material for: Idiopathic Premature Ventricular Contraction Catheter Ablation, Sedentary Population vs. Athlete’s Populations: Outcomes and Resumption of Sports Activity
Source: J Clin Med. 2024 Mar 24;13(7):1871. doi: 10.3390/jcm13071871 (PMC11012949; doi:10.3390/jcm13071871)
Supplement: Supplementary file 1 [file jcm-13-01871-s001.zip › jcm-2842894-supplementary.pdf]

Supplementary Materials

Definition:

- Sedentary patient: a patient who does not engage in regular and habitual physical activity
- Athletes patient: a patient who engage in regular and habitual physical activity
- Agonist athletes patient: patients who engage in sports physical activity not recreationally but at high intensity level aimed at competition.

RVOT, LVOT and fascicular origin from 12-lead-ECG was established in relation to the criteria proposed by the these articles: 24,25,26.
